# Associations Between CSF Markers of Inflammation, White Matter Lesions, and Cognitive Decline in Individuals Without Dementia

**DOI:** 10.1212/WNL.0000000000207113

**Published:** 2023-04-25

**Authors:** Eske Christiane Gertje, Shorena Janelidze, Danielle van Westen, Nicholas Cullen, Erik Stomrud, Sebastian Palmqvist, Oskar Hansson, Niklas Mattsson-Carlgren

**Affiliations:** From the Clinical Memory Research Unit (E.C.G., S.J., N.C., E.S., S.P., O.H., N.M.-C.), Department of Clinical Sciences Malmö, Lund University; Department of Internal Medicine (E.C.G.), Skåne University Hospital, Lund; Diagnostic Radiology (D.v.W.), Department of Clinical Sciences Lund, Lund University; Imaging and Function (D.v.W.), Skåne University Hospital, Lund; Memory Clinic (N.C., N.M.-C.), Skåne University Hospital, Malmö; Department of Clinical Sciences Lund, Neurology (E.S., S.P., O.H.), Lund University, Skåne University Hospital; and Wallenberg Center for Molecular Medicine (N.M.-C.), Lund University, Sweden.

## Abstract

**Background and Objectives:**

Small vessel disease (SVD) and neuroinflammation both occur in Alzheimer disease (AD) and other neurodegenerative diseases. It is unclear whether these processes are related or independent mechanisms in AD, especially in the early stages of disease. We therefore investigated the association between white matter lesions (WML; the most common manifestation of SVD) and CSF biomarkers of neuroinflammation and their effects on cognition in a population without dementia.

**Methods:**

Individuals without dementia from the Swedish BioFINDER study were included. The CSF was analyzed for proinflammatory markers (interleukin [IL]–6 and IL-8), cytokines (IL-7, IL-15, and IL-16), chemokines (interferon γ–induced protein 10, monocyte chemoattractant protein 1), markers of vascular injury (soluble intercellular adhesion molecule 1, soluble vascular adhesion molecule 1), and markers of angiogenesis (placental growth factor [PlGF], soluble fms-related tyrosine kinase 1 [sFlt-1], vascular endothelial growth factors [VEGF-A and VEFG-D]), and amyloid β (Aβ)42 Aβ40, and p-tau217. WML volumes were determined at baseline and longitudinally over 6 years. Cognition was measured at baseline and follow-up over 8 years. Linear regression models were used to test associations.

**Results:**

A total of 495 cognitively unimpaired (CU) elderly individuals and 247 patients with mild cognitive impairment (MCI) were included. There was significant worsening in cognition over time, measured by Mini-Mental State Examination, Clinical Dementia Rating, and modified preclinical Alzheimer composite score in CU individuals and patients with MCI, with more rapid worsening in MCI for all cognitive tests. At baseline, higher levels of PlGF (β = 0.156, *p* < 0.001), lower levels of sFlt-1 (β = −0.086, *p* = 0.003), and higher levels of IL-8 (β = 0.07, *p* = 0.030) were associated with more WML in CU individuals. In those with MCI, higher levels of PlGF (β = 0.172, *p* = 0.001), IL-16 (β = 0.125, *p* = 0.001), IL-8 (β = 0.096, *p* = 0.013), IL-6 (β = 0.088, *p* = 0.023), VEGF-A (β = 0.068, *p* = 0.028), and VEGF-D (β = 0.082, *p* = 0.028) were associated with more WML. PlGF was the only biomarker that was associated with WML independent of Aβ status and cognitive impairment. Longitudinal analyses of cognition showed independent effects of CSF inflammatory markers and WML on longitudinal cognition, especially in people without cognitive impairment at baseline.

**Discussion:**

Most neuroinflammatory CSF biomarkers were associated with WML in individuals without dementia. Our findings especially highlight a role for PlGF, which was associated with WML independent of Aβ status and cognitive impairment.

White matter lesions (WML) are part of the small vessel disease (SVD) spectrum and associated with cognitive decline and dementia.^1,2^ They may be caused by reduced cerebral blood flow and loss of autoregulation resulting in chronic and diffuse subclinical ischemia, causing demyelination and axonal loss.^3^ The etiology of SVD is not fully understood. Previous studies suggest a role of endothelial dysfunction in SVD, especially in WML.^4,5^

The pathophysiologic mechanisms of neurodegenerative diseases involve changes in neuronal and non-neuronal cells in the brain, for example, in microglia and astrocytes, which modulate immunologic responses and neuroinflammation.^6-8^ Neuroinflammation is linked to impairment of the blood-brain barrier (BBB), which is part of the neurovascular unit.^9^ Disruption of the BBB can be observed in neurodegenerative diseases, including Alzheimer disease (AD), and in cerebrovascular disease. It can be induced by hypoxia or ischemia.^10-12^

CSF biomarkers of neuroinflammation and cerebrovascular dysfunction are associated with cognitive decline.^9,13,14^ According to previous studies, markers of endothelial dysfunction are more strongly associated with SVD than those of systemic inflammation.^15^ Associations between vascular endothelial growth factor (VEGF-A) and placental growth factor (PlGF) with WML have been reported in patients with Parkinson disease.^16^ In addition, plasma VEGF-D correlated with greater cerebral SVD burden in individuals without dementia or stroke.^15,17^ However, it is unclear to what degree neuroinflammatory CSF biomarkers are correlated with vascular pathology in people with or without AD pathology.

We investigated the relationship between SVD and neuroinflammation and their impact on cognition by studying the association between WML, CSF biomarkers of inflammation, and cognitive tests. Our primary aim was to estimate the effect of inflammation on WML, both cross-sectionally and longitudinally. We hypothesized that markers involved in endothelial dysfunction such as soluble intercellular adhesion molecule 1 (sICAM-1) and soluble vascular adhesion molecule 1 (sVCAM-1)^5^ and markers of the VEGF family are associated with WML.^9,13,18^ Reduced blood flow and hypoxia due to SVD such as WML might trigger angiogenesis (which is associated with the upregulation of markers of the VEGF family).^17,19^ Findings from previous studies have been inconsistent on associations between proinflammatory markers and WML.^15,16^ In this study, we tested a specific hypothesis that proinflammatory markers such as interleukin (IL)–6 and IL-8 are associated with WML. Last, we hypothesized that a combination of neuroinflammation and cerebrovascular injury increases the risk of more aggressive neurodegenerative diseases, such as AD. Our secondary aim was therefore to test whether interactions between inflammatory markers and WML predict longitudinal cognitive decline and whether there are differences depending on β-amyloid (Aβ) status (indicating the presence of AD brain changes). In symptomatic patients, we also stratified by tau status.

## Methods

### Study Population

In this cohort study, cognitively unimpaired (CU) elderly individuals, patients with subjective cognitive decline (SCD), and patients with mild cognitive impairment (MCI) were included. CU individuals were recruited from the population-based Malmö Diet Cancer Study, and patients with SCD and MCI were enrolled at 3 memory outpatient clinics between 2010 and 2014 as part of the Swedish BioFINDER study (biofinder.se).^13^ Inclusion criteria for cognitively healthy controls were (1) age 60 years or older, (2) Mini-Mental State Examination (MMSE) 28–30 points at the screening visit, (3) absence of cognitive symptoms as evaluated by a physician, (4) fluent in Swedish, and (5) not fulfilling the criteria of MCI or any dementia. Exclusion criteria were (1) significant neurologic or psychiatric disease (e.g., stroke, Parkinson disease, multiple sclerosis, and major depression), (2) significant systemic illness making it difficult to participate, (3) significant alcohol abuse, or (4) refusing lumbar puncture. Patients with SCD and MCI were thoroughly examined by physicians specialized in dementia disorders. Inclusion criteria for patients with SCD and MCI were (1) cognitive symptoms, (2) not fulfilling the criteria for dementia, (3) MMSE 24–30 points, (4) age 60–80 years, and (5) fluent in Swedish. Patients with (1) cognitive impairment that without doubt could be explained by another condition (other than prodromal dementia), (2) severe somatic disease, and (3) refusing lumbar puncture or neuropsychological investigation were excluded. Patients with SCD vs MCI were assessed with a neuropsychological battery assessing the cognitive domains of verbal ability, visuospatial construction, episodic memory, and executive functions and the clinical assessment of a senior neuropsychologist.^20^ In agreement with guidelines, cognitively normal individuals and study participants with SCD were included in the CU group.^13,21^

### CSF Collection and Analysis

CSF samples were collected and handled according to a standardized protocol.^22^ Samples were taken from nonfasting individuals by lumbar puncture at 3 different centers. They were centrifuged (2,000*g*, +4°C, 10 minutes), 1 mL was aliquoted into polypropylene tubes (Sarstedt AG & Co, Nümbrecht, Germany), and aliquots were stored at −80°C. Samples went through 1 freeze-thaw cycle before the analysis when 200 μL was further aliquoted into LoBind tubes (Eppendorf Nordic A/S, Hørsholm, Denmark).

An ultrasensitive Mesoscale Discovery immunoassay and a customized V-PLEX kit were used to analyze CSF concentrations of proinflammatory markers (IL-6 and IL-8), cytokines (IL-7, IL-15, and IL-16), chemokines (interferon γ–induced protein 10 [IP-10], monocyte chemoattractant protein 1 [MCP-1]), markers of vascular injury (sICAM-1 and sVCAM-1), and markers of angiogenesis (PlGF, soluble fms-related tyrosine kinase 1 [sFlt-1], VEGF-A, and VEGF-D), as previously described^13^ (Table 1). Assays were selected from the preconfigured V-PLEX Neuroinflammation Panel 1 Human kit (combining proinflammatory, cytokine, chemokine, and angiogenesis panels), restricted to analytes with intra-assay and interassay coefficients of variation below 20% and to assays sensitive enough for CSF analysis in test runs. Samples were analyzed with the customized kit according to the manufacturer's recommendations with 1 modification: for chemokine and proinflammatory panels, samples and calibrators were incubated overnight at +4°C. CSF concentrations of Aβ42 and Aβ40 were measured using ELISA kits according to the manufacturer's recommendations (Aβ42, Aβ40; EUROIMMUN AG, Lübeck, Germany). All analyses were performed using 1 batch of reagents. Samples were randomized across plates/runs to minimize effects of run-to-run variation.^13^ CSF p-tau217 was measured using a Mesoscale Discovery immunoassay developed by Lilly Research Laboratories. Samples were analyzed as previously described for plasma samples.^23^ For CSF analysis, we used a different calibrator range and 1:4 sample dilution.

**Table 1:**
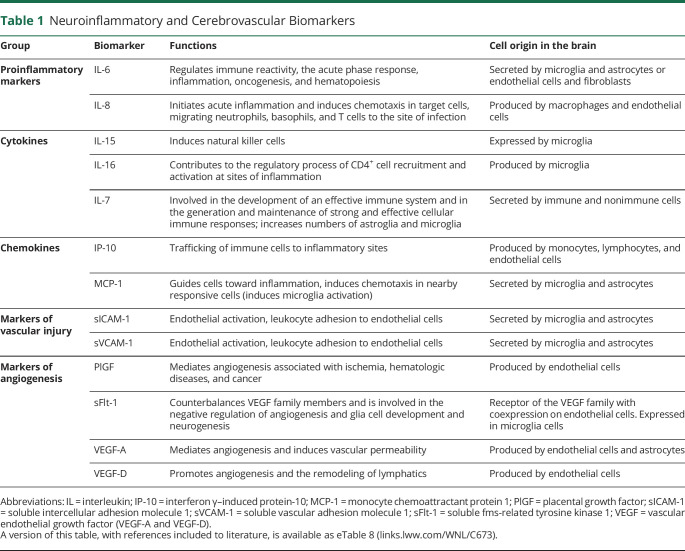
Neuroinflammatory and Cerebrovascular Biomarkers

### MRI

MRI was performed with a 3T Siemens (Erlangen, Germany) Trio system equipped with a standard 12-channel head coil. The protocol comprised an axial T2 fluid-attenuated inversion recovery imaging and sagittal MPRAGE sequence. Automated segmentation of WML was performed using the Lesion Segmentation Tool implemented in SPM8; this generated a total lesion volume (named WML volume) for each individual. The hippocampal volume (HCV) (total HCV: [right HCV + left HCV]/2) and the intracranial volume (ICV) were determined by FreeSurfer (version 5.3) using the MPRAGE images. Longitudinal follow-up was performed with MRI after 2, 4, and 6 years from baseline.

### Cognitive Testing

Cognitive assessment included the MMSE,^24^ global Clinical Dementia Rating (CDR),^25^ the 10-word delayed recall test from the Alzheimer Disease Assessments scale—cognitive subscale (ADAS-cog),^26^ the Trail Making Test B (TMTB),^27^ and the animal and letter S fluency tests.^28^ A modified preclinical Alzheimer composite score (mPACC) was calculated based on the average of *z* scores from different tests (mPACC = [MMSE (*z*) + 2 × ADAS-cog (*z*) + animal fluency (*z*) + TMTB (*z*)]/5). The ADAS-cog was counted twice to preserve the weight of memory from the original PACC.^29,30^ Tests were repeated after 2, 4, 6, and 8 years for CU individuals and after 1, 2, 3, 4, 5, and 8 years for patients with MCI and SCD.

### Statistical Analyses

The subgroups CU and MCI were analyzed separately because these groups may differ regarding brain and cognitive changes. All participants had WML data, and data on at least 1 CSF biomarker (a list of missing data is detailed in eTable 1, links.lww.com/WNL/C673). CU and MCI baseline characteristics were compared using the Student *t* test (continuous variables) or χ^2^ test (categorical variables). Variables that were not normally distributed (IP-10, IL-6, IL-8, and WML volume) were log10 transformed. CSF biomarker and WML volume data were standardized to *z* scores based on the entire population when used as predictors. In accordance with definitions on the Alzheimer continuum, following definitions from the National Institute of Aging-Alzheimer Association,^21^ study participants were categorized into groups with normal (Aβ−) or pathologic (Aβ+) CSF signature using the CSF Aβ42/Aβ40 ratio (cutoff ≤0.1). Aβ+ were sufficient to indicate that individuals were on the Alzheimer continuum.^31^ In a sensitivity analysis, we validated results in a further specified MCI group, including those that were positive for Aβ and a tau biomarker (A+T+). For T+, we used CSF p-tau217, which has been shown to be highly correlated to tau pathology in the brain.^32^ We derived a cutoff value from p-tau217 at the mean +2 SD in Aβ− CU individuals of a larger population from the BioFINDER study (n = 403). Using this cutoff, our MCI population was divided into A−T− MCI (N = 93), A−T+ MCI (N = 8), A+T− MCI (N = 17), and A+T+ MCI (N = 113).

Statistical analyses were conducted in 3 steps. First, associations between baseline CSF neuroinflammatory biomarkers (used as predictors) and baseline WML volume (used as outcome) were tested in linear regression models for each neuroinflammatory biomarker separately, univariately, and multivariately (including covariates age, gender, ICV, and Aβ status). In a sensitivity analysis, we also added CSF Aβ40 as an additional covariate to the models to correct for individual differences in CSF production.^33^ We then evaluated vascular risk factors as possible confounding factors. All vascular risk factors that were univariately associated with WML in the subgroups of CU and MCI were included as covariates for the association between CSF biomarkers and WML. Regression analyses showed no evidence of multicollinearity. Next, a multivariable regression analysis was performed including all univariately significant neuroinflammatory markers and covariates in the same model. Then, associations between neuroinflammatory markers and baseline WML were analyzed by Aβ status.

Associations between baseline CSF biomarkers, covariates, and longitudinal WML volumes were analyzed using linear mixed-effect (LME) models, with age, gender, ICV, and Aβ status as covariates, including random intercepts and slopes. Third, the effect of baseline neuroinflammatory markers and baseline WML volumes on longitudinal changes in cognition (MMSE, global CDR, and mPACC) was tested. LME models were used to test longitudinal changes in cognitive scores and differences between diagnostic groups and then to test for the effects of WML and neuroinflammatory markers on cognitive changes in separate models and in models that included both (and their interaction with time) simultaneously. All models were adjusted for age, gender, education, ICV, HCV, and Aβ status, including random intercepts and slopes. Adjustment for multiple comparisons was performed using the false discovery rate (FDR) according to the Benjamini-Hochberg procedure; *p*_FDR_ < 0.05, indicating statistical significance for all regression models and LME models. All individuals with available data were included in linear regression models and LME models, except 1 outlier on PlGF, which was excluded from analyses including PlGF concentration. Statistical analyses were performed using SPSS software, version 26 (SPSS Statistics for 240 Windows, version 24.0; IBM Corp., Armonk, NY), and R (version 4.0.0).

### Standard Protocol Approvals, Registrations, and Patient Consents

The study has been approved by the Regional Ethic Committee at Lund University, Sweden (Dnr 2010/156, and Dnr 695/2008). All participants gave written informed consent to participate in the study. Methods were performed in accordance with the approved guidelines.

### Data Availability

By request from a qualified academic investigator, anonymized data will be shared for the sole purpose of replicating procedures and results presented in the article. The data transfer needs to be in agreement with the EU legislation on the general data protection regulation and decisions by the Ethical Review Board of Sweden and Region Skåne, which should be regulated in a material transfer agreement.

## Results

### Demographics

The study population included 495 CU individuals and 247 patients with MCI. Baseline characteristics are summarized in Table 2. The CU group was older (*t* = 2.66, *p* = 0.008), had more females (χ(1) = 27.85, *p* < 0.001), longer education (*t* = 4.10, *p* < 0.001), more cases with hyperlipidemia (χ(1) = 45.23, *p* < 0.001), higher MMSE scores (*t* = 15.76, *p* < 0.001), less ICV (*t* = −5.55, *p* < 0.001), and larger HCV (*t* = 6.24, *p* < 0.001) than the MCI group. The MCI group had more Aβ positive individuals (χ(1) = 54.47, *p* < 0.001), more patients with a stroke (χ(1) = 24.99, *p* < 0.001) and ischemic heart disease (IHD) (χ(1) = 12.39, *p* < 0.001), and more WML (*t* = −7.30, *p* < 0.001) than CU. The A+T+ MCI group did not differ from the A+ MCI group on any covariate.

**Table 2:**
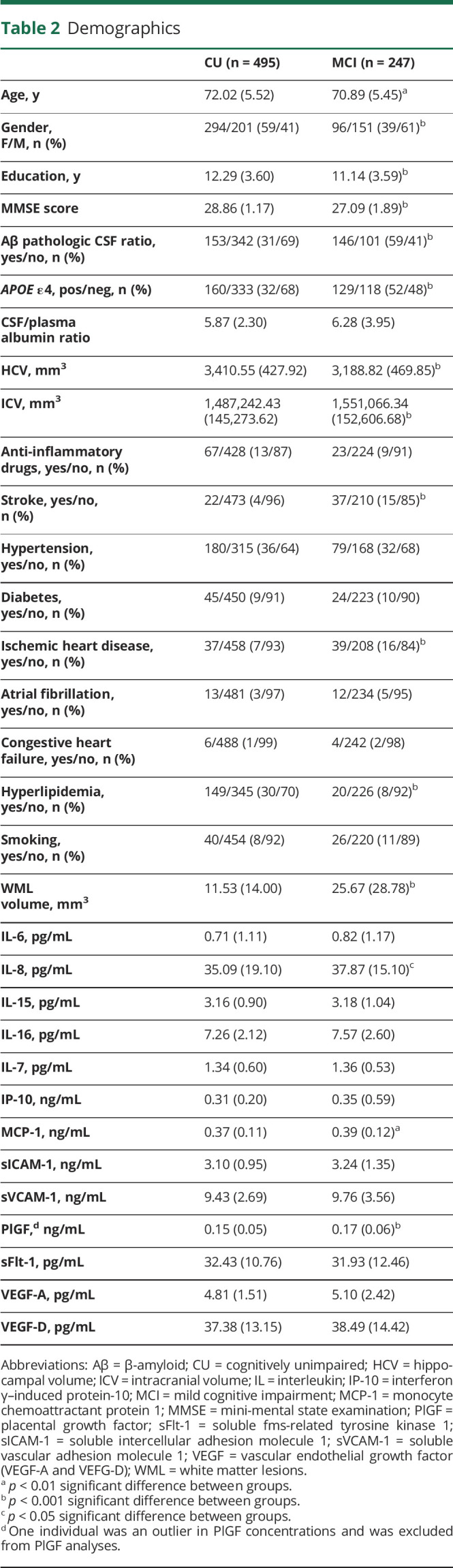
Demographics

### Associations Between CSF Neuroinflammatory Markers and WML at Baseline

**Figure 1:**
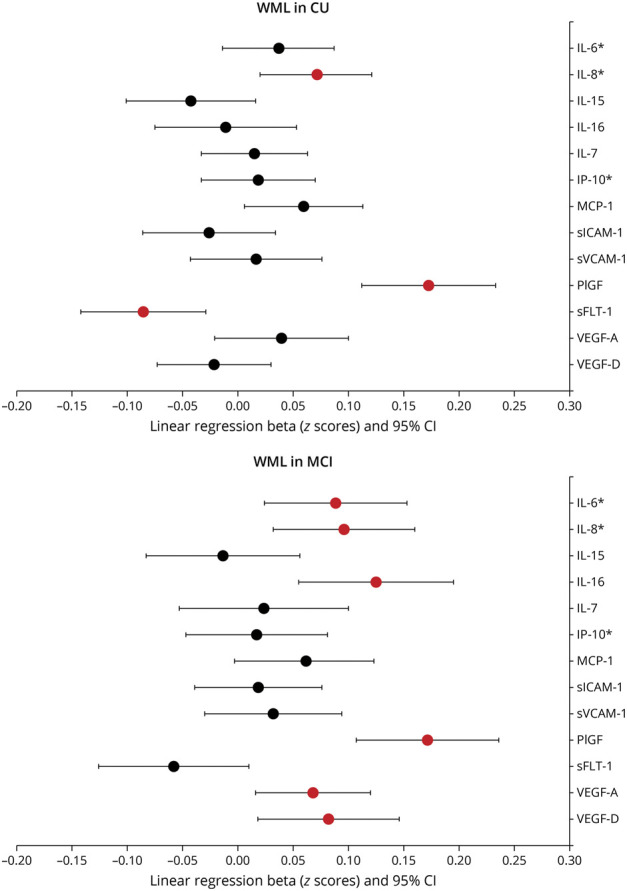
Associations Between Different Neuroinflammatory Markers and WML Summarized in Forest Plots for CU Individuals and Patients With MCI Aβ = β-amyloid; CU = cognitively unimpaired; IL = interleukin; IP-10 = interferon γ–induced protein-10; MCI = mild cognitive impairment; MCP-1 = monocyte chemoattractant protein 1; PlGF = placental growth factor; sFlt-1 = soluble fms-related tyrosine kinase 1; sICAM-1 = soluble intercellular adhesion molecule 1; sVCAM-1 = soluble vascular adhesion molecule 1; VEGF = vascular endothelial growth factor (VEGF-A and VEFG-D); WML = white matter lesions. *log10 transformed. RED = significant after correction for multiple comparison with *p* < 0.05. Linear regression models were adjusted for age, gender, intracranial volume, and CSF Aβ status, and all *p* values were corrected for multiple comparison.

In univariate analyses, age, gender, and ICV were significantly associated with WML, but there were no associations for CSF Aβ status. In multivariable analyses, associations for age and ICV with WML remained stable, while associations for gender and WML were attenuated (eTable 2, links.lww.com/WNL/C673). In CU individuals, higher levels of IL-8 (β = 0.07, *p* = 0.03) were associated with greater WML volume in adjusted models (every SD higher level of IL-8 was associated with 0.07 mm^3^ increase in log 10 of WML volume). Higher levels of PlGF (β = 0.17, *p* = 0.001), and lower levels of sFlt-1 (β = −0.09, *p* = 0.02) were also associated with greater WML volume. In the MCI group, higher levels of IL-6 (β = 0.09, *p* = 0.023), IL-8 (β = 0.10, *p* = 0.013), IL-16 (β = 0.13, *p* = 0.001), PlGF (β = 0.17, *p* = 0.001), VEGF-A (β = 0.07, *p* = 0.028), and VEGF-D (β = 0.08, *p* = 0.028) were associated with greater WML volume (Figure 1).

In a sensitivity analysis, CSF Aβ40 was added to the model as an additional covariate. Most of the associations were not affected by this additional adjustment. But, in CU individuals, the association for IL-16 (β = 0.16, *p* = 0.001), MCP-1 (β = 0.05, *p* = 0.048), VEGF (β = 0.07, *p* = 0.021), and VEGF-D (β = 0.11, *p* = 0.003) became significant, while the association for sFlt-1(β = −0.01, *p* = 0.906) was lost. In patients with MCI, the associations for IL-6 (β = 0.03, *p* = 0.431), IL-16 (β = 0.02, *p* = 0.818), VEGF-A (β = 0.04, *p* = 0.431), and VEGF-D (β = 0.00, *p* = 0.972) were lost, while those for PlGF and IL-8 remained significant (eTable 3, links.lww.com/WNL/C673).

In a further sensitivity analysis, we tested the associations between CSF biomarkers and WML when adjusting for potential confounding vascular risk factors. Atrial fibrillation, hypertension, and stroke were associated with WML in CU individuals, whereas diabetes and stroke were associated with WML in those with MCI (Pearson correlation tests, *p* < 0.05, *r* coefficients 0.1–0.3). Therefore, these vascular risk factors were added as additional covariates to the main models. The associations with WML remained significant with this adjustment in CU individuals for IL-8, PlGF, and sFlt-1. In patients with MCI, associations remained significant for IL-6, IL-8, PlGF, and VEGF-A (all *p* < 0.05), but the association between VEGF-D and WML was attenuated (*p* = 0.074). In those models, stroke (but not the other vascular risk factors) remained significantly associated with WML.

### Independent Effects of CSF Neuroinflammatory Markers on Baseline WML

Significant biomarkers from univariate analyses were used simultaneously as predictors together with the covariates. In CU individuals, PlGF (β = 0.17, *p* < 0.001), IL-8 (β = 0.08, *p* = 0.009), and sICAM-1 (β = −0.10, *p* = 0.022) remained significant (*R*^2^ = 0.36), meaning that 36% of WML volume can be explained by the predicting biomarker variables and the covariates, with PlGF here being the strongest predictive biomarker. In patients with MCI, PlGF (β = 0.15, *p* < 0.001), IL-16 (β = 0.15, *p* = 0.002), and sICAM-1 (β = −0.11, *p* = 0.030) remained significant (*R*^2^ = 0.36). The *R*^2^ for each marker separately is summarized in eTable 4 (links.lww.com/WNL/C673).

### CSF Neuroinflammatory Markers and Baseline WML by Aβ Status

**Figure 2:**
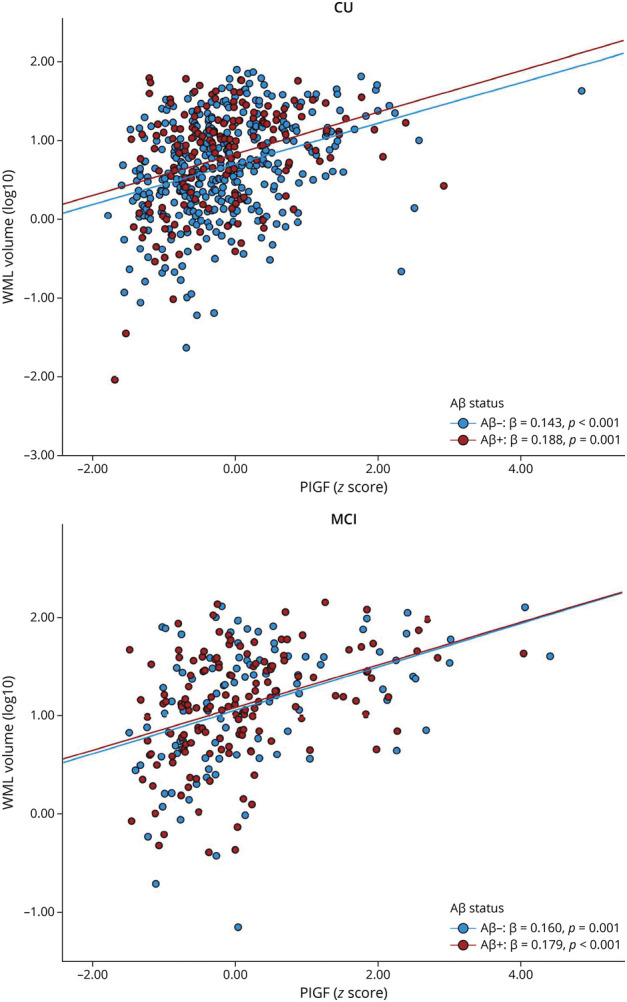
PlGF Is Associated With WML in CU Individuals and Patients With MCI Independent of Cognitive Impairment and Aβ Status Aβ = β-amyloid; CU = cognitively unimpaired; MCI = mild cognitive impairment; PlGF = placental growth factor; WML = white matter lesions. Linear regression models were adjusted for age, gender, and intracranial volume and all *p* values were corrected for multiple comparison.

Finally, associations between neuroinflammatory markers and baseline WML were analyzed by Aβ status (eTable 5, links.lww.com/WNL/C673). Higher PlGF was associated with more WML across all subgroups independent of Aβ status and cognitive state (Figure 2): Aβ+ CU PlGF (β = 0.19, *p* = 0.013), Aβ− CU PlGF (β = 0.16, *p* = 0.001), Aβ+ MCI PlGF (β = 0.18, *p* = 0.001), and Aβ− MCI PlGF (β = 0.16, *p* = 0.007). In Aβ + MCI, this association remained significant, when restricting to the A+T+ MCI group (β = 0.17, *p* = 0.026) in a sensitivity analysis. Furthermore, in Aβ− CU, lower sFlt-1(β = −0.12, *p* = 0.039) was associated with more WML and in Aβ− MCI, higher IL-8 (β = 0.14, *p* = 0.039), higher IL-16 (β = 0.18, *p* = 0.009), and higher VEGF-A (β = 0.18, *p* = 0.007) were associated with more WML (eFigure 1).

### CSF Neuroinflammatory Markers and Longitudinal WML Volume

Our data showed an increase in WML over time, both in individuals with low and high WML burden at baseline (eFigure 2, links.lww.com/WNL/C673). When using longitudinal WML as outcome, lower PlGF (β = −0.01, *p* = 0.021) was associated with increasing WML over time in CU individuals. In patients with MCI, lower IL-6 (β = −0.01, *p* = 0.028), IL-8 (β = −0.01, *p* = 0.033), IL-16 (β = −0.01, *p* = 0.028), MCP-1 (β = −0.01, *p* = 0.052), PlGF (β = −0.01, *p* = 0.004), and VEGF-A (β = −0.01, *p* = 0.028) were associated with increasing WML over time. We also tested these analyses when stratifying by Aβ status. Associations between lower PlGF and increase in WML volume were seen in Aβ+ CU (β = −0.02, *p* = 0.032) and Aβ+ MCI (β = −0.03, *p* = 0.031), but not in Aβ− subgroups. Greater age and ICV were associated with more longitudinal WML (*p* < 0.05) in subgroups, both when including biomarkers and when used alone without biomarkers. Female sex and positive Aβ status were associated with more longitudinal WML in CU individuals (*p* < 0.05) in both models, but not in MCI. The effect of positive Aβ status on WML in CU individuals was stable in models without biomarker (β = 0.012, *p* = 0.014) and when used together with PlGF (β = 0.011, *p* = 0.025). In CU individuals, PlGF had no effect in the unadjusted model due to the lack of ICV as a covariate (eTable 6).

### CSF Neuroinflammatory Markers and Baseline WML Predicting Longitudinal Cognitive Decline Separately

Cognition generally declined over time in both groups (eFigure 3, links.lww.com/WNL/C673). A decline in MMSE was observed in CU individuals (β = −0.26, *p* < 0.001, meaning a decline in 0.26 units per year), with a steeper decline in patients with MCI (group × time interaction term: β = −1.39, *p* < 0.001, meaning that MMSE declined 1.39 units more per year in patients with MCI than in CU individuals, on average). For CDR, an increase (indicating worse cognition) over time in the CU group (β = 0.03, *p* < 0.001) with a steeper increase in the MCI group (group × time: β = 0.16, *p* < 0.001) was observed. Cognitive decline was also seen in mPACC in CU individuals (β = −0.08, *p* < 0.001) with a steeper decline in patients with MCI (group × time in: β = −0.27, *p* < 0.001).

We then tested associations between neuroinflammatory markers, WML volume, and longitudinal MMSE score. When testing for the univariate effect in subgroups, no associations were found between neuroinflammatory markers and longitudinal MMSE. However, greater baseline WML volume was associated with longitudinal decline in MMSE in CU (β = −0.11, *p* = 0.001), and MCI (β = −0.19, *p* = 0.028) (Table 3).

**Table 3:**
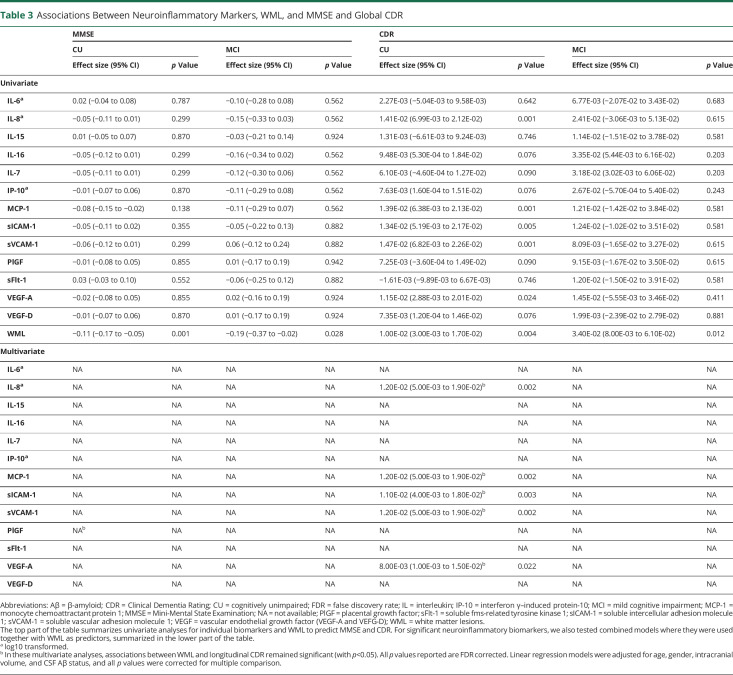
Associations Between Neuroinflammatory Markers, WML, and MMSE and Global CDR

Next, we tested global CDR as outcome. In the CU group, associations were seen between higher IL-8 (β = 0.01, *p* = 0.001), MCP-1 (β = 0.01, *p* = 0.001), sICAM-1 (β = 0.01, *p* = 0.005), sVCAM-1 (β = 0.01, *p* = 0.001), and VEGF-A (β = 0.01, *p* = 0.024) and increased longitudinal global CDR. No associations were seen in the MCI group. Associations between more WML and higher longitudinal CDR were seen in CU (β = 0.01, *p* = 0.004), and MCI (β = 0.03, *p* = 0.012) (Table 3).

No associations were seen between neuroinflammatory markers and mPACC in any groups. Main effects between more WML and declining mPACC in CU individuals (β = −0.03, *p* < 0.001) but not in patients with MCI were found (eTable 7, links.lww.com/WNL/C673).

### Combining CSF Neuroinflammatory Markers and Baseline WML to Predict Longitudinal Cognitive Decline

We then tested to what degree associations in CU individuals for WML and neuroinflammatory markers with CDR were independent. For all neuroinflammatory markers that were significant univariately, we tested models that also included WML (and its interaction with time) as an additional predictor of longitudinal decline. Associations for both, biomarkers and WML, with longitudinal CDR in CU individuals remained significant, when adjusting for the other modality (Table 3). These analyses were not relevant for MMSE or mPACC because there were no univariate associations for neuroinflammatory biomarkers and longitudinal MMSE or mPACC in the groups.

## Discussion

**Figure 3:**
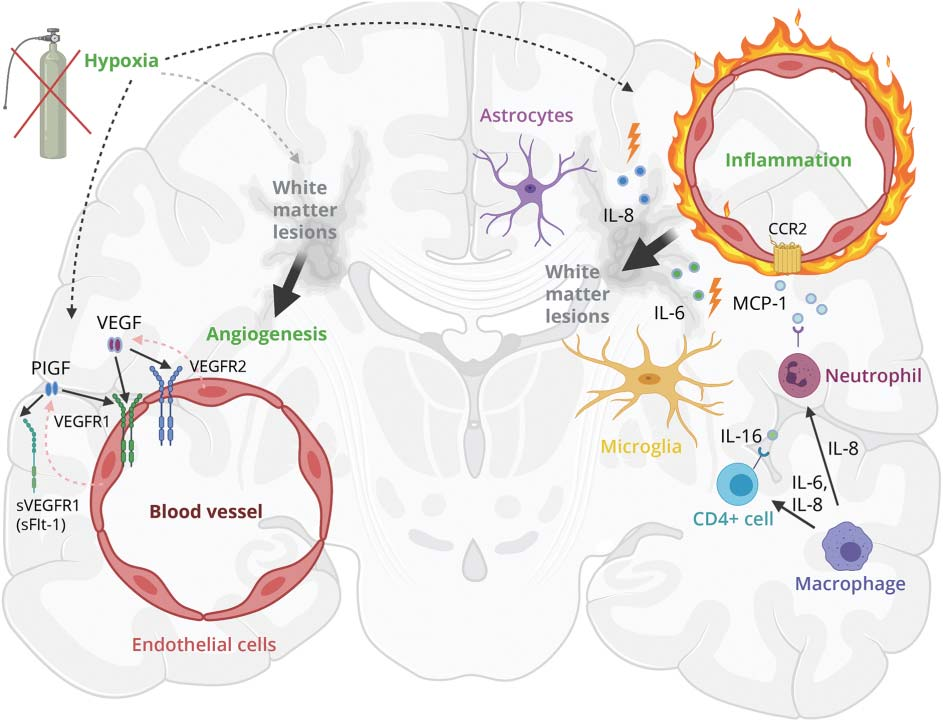
Theoretical Model of the Interaction Between WML and Markers of Neuroinflammation and Angiogenesis Based on Current Results and Review of the Literature Hypoxia induces neuroinflammation and endothelial cells, microglia, and astrocytes release proinflammatory markers IL-8 and IL-6 and IL-16 and MCP-1. Those cytokines could be involved in WML pathology by BBB disruption and disruption of the neurovascular unit, resulting in gliosis and thus WML.^19^ Hypoxia and neuroinflammation could stimulate upregulation of PlGF and VEGF, inducing pathologic angiogenesis in damaged white matter in the acute phase.^11,19,48^ This original figure was designed for this article and created with BioRender.com. IL = interleukin; MCP-1 = monocyte chemoattractant protein 1; PlGF = placental growth factor; sVEGFR1 = soluble VEGF receptor 1 (same as sFlt-1); VEGF = vascular endothelial growth factor (VEGF-A and VEFG-D); VEGFR1/VEGFR2 = VEGF receptor 1/2; WML = white matter lesions.

This study investigated associations between CSF biomarkers of neuroinflammation and cerebrovascular dysfunction, SVD, and longitudinal cognitive decline, in both CU individuals and patients with MCI. The proinflammatory markers IL-8 and biomarkers of cerebrovascular dysfunction such as PlGF and sFlt-1 were associated with greater WML volume in CU individuals. In patients with MCI, not only IL-8 and PlGF but also IL-6, IL-16, VEGF-A, and VEGF-D were significantly associated with greater WML volume. We noted that the general levels of WML were greater in patients with MCI than in CU individuals, which may increase the power to detect associations with neuroinflammatory biomarkers. Associations between higher levels of serum IL-8 and WML in cognitively impaired no dementia and AD have been previously described,^34^ and the involvement of IL-8, sFlt-1, and VEGF-A in BBB impairment has also been previously described.^9,13,18^ We therefore suggest that upregulation of those cytokines could be involved in BBB impairment and contribute to the progress of WML (Figure 3). IL-16 is a cytokine, which may be involved in cell recruitment and activation at sites of inflammation. It seems to increase in areas of neurodegeneration and inflammation in the brain.^35^ In a recent study, a higher trend of CSF IL-16 concentration was observed in BBB impairment (defined as CSF albumin index ≥9).^9^

According to other studies, the proinflammatory markers IL-8 and IL-6 are produced by microglia, astrocytes, and endothelial cells and could be involved in stimulating growth factor production.^16,36^ In our study, elevated concentrations of different members of the VEGF family were associated with greater WML volume. PlGF and VEGF-A are believed to mediate angiogenesis, regulate vascularization, and induce vascular permeability.^37,38^ Our findings suggest that markers of the VEGF family, especially PlGF, may be involved in the pathophysiology around WML, possibly by its contribution to vascular permeability and neuroinflammation.^37^ When examining groups based on the presence of the Alzheimer continuum (Aβ positive vs Aβ negative) separately, the above described inflammatory makers were only associated with more WML in Aβ negative subgroups, except for PlGF. Most strikingly, higher PlGF levels were associated with greater WML volumes across all groups, independently of Aβ status and cognitive impairment, supporting converging pathways between neuroinflammation and cerebrovascular pathology. Therefore, PlGF seems to be a general marker of WML. Of interest, Winder et al.^39^ found that plasma PlGF and VEGF were associated with cerebral amyloid angiopathy, another marker of SVD that was obtained by postmortem neuropathologic examination. This supports our findings that there is an association between processes of angiogenesis and SVD. PlGF upregulation can be induced by different stimuli including hypoxia, other inflammatory cytokines, growth factors, or oncogenes.^40^ Compared with VEGF-A (which binds to VEGFR-1 receptor and VEGFR-2 receptor), PlGF only binds to VEGFR-1 receptor, which is considered the main signaling receptor in angiogenesis.^40^ The soluble receptor isoform of VEGFR-1, sFlt-1, which can be spliced from VEGFR-1 can bind and inhibit the action of PlGF and VEGF-A, followed by reduced blood vessel growth. Our results showed associations between lower concentrations of sFlt-1 (which is believed to counterbalance PlGF) and WML, suggesting that it is possible that there is increased angiogenesis in individuals with more WML (Figure 3).

Growth factors of the VEGF family play an important role in protection and recovery after ischemia, regulating angiogenesis, and neurogenesis.^41,42^ Our longitudinal results unexpectedly showed that lower levels of PlGF were associated with more rapid longitudinal WML volume increase in both groups, with a slightly stronger association in MCI. In addition, lower levels of IL-6, IL-8, IL-16, MCP-1, and VEGF-A were associated with longitudinal increase in WML in patients with MCI. This was a surprising finding because we expected that higher biomarker levels would be associated with more WML over time. Dobrynina et al.^43^ described lower VEGF-A levels at baseline being associated with more WML in a group of patients with increased prevalence of periventricular WML and more atrophy in general. One possibility is that there are associations between widespread vascular wall damage destroying endothelial cells and lower growth factor production.^43,44^ This could then be followed by a decline in cerebrovascular angiogenesis resulting in hypoxia-induced capillary loss and more WML.^45^ In a postmortem analysis, decreased concentrations of several cytokines including IL-16, IL-8, and IL-6 in the frontal white matter across different dementia groups compared with those in controls without dementia were described.^46^ Venous collagenosis—which is associated with WML—is believed to cause venous insufficiency, resulting in vessel leakage and vasogenic edema, which may contribute to dynamic changes in WML volumes over time.^47^ Taken together, the literature may be in agreement with our observation of associations between lower biomarker levels and increased WML over time. The magnitudes of the associations between biomarkers and longitudinal WML in our study were small. Because there were associations at baseline between higher biomarker levels and more WML, the longitudinal results may also partly be caused by a regression to the mean phenomenon. We also analyzed the effect of covariates in univariate analyses. We conclude that positive Aβ status predicts more WML over time in CU individuals (but not in patients with MCI), in a manner that seems to be independent of the biomarkers tested in this study. Considering all the above, the associations between biomarkers and longitudinal WML need to be validated in an independent cohort.

Finally, we analyzed independent contributions of inflammatory markers and WML to predict longitudinal cognitive decline. IL-8, IL-15, MCP-1, sICAM-1, sVCAM-1, sFlt-1, and VEGF-A have been previously described to be associated with cognitive decline and may be involved in BBB impairment.^9,13,18^ We found that the effects of WML on cognitive decline were independent of biomarker levels. Some biomarkers (IL-8, MCP-1, sICAM-1, sVCAM-1, and VEGF-A) had independent effects on cognition in the CU group even when adjusting for WML, suggesting that this represents a partly distinct pathway leading to cognitive decline.

The strengths of this study include a large sample size of CU individuals and patients with MCI and the multimodal design with a broad panel of neuroinflammatory markers. Studies with these neuroinflammatory markers in the CSF in relation to vascular pathology are rare. Previous studies mainly focused on plasma measurements, which may be less accurate for changes in the CNS.^17,39^ A weakness is the lack of patients with a diagnosis of vascular dementia. Because the cohort was recruited from a Swedish memory clinic (mainly of European ancestry), results may not be completely generalizable to the general population or people with other ethnical backgrounds. In addition, it is possible that different pathologic processes in the brain may affect CSF levels of neuroinflammatory markers. We tried to overcome this by adjusting for different relevant factors (including biomarkers representing both Aβ pathology, and in MCI, also tau pathology). We adjusted associations between biomarkers and WML for possible confounding vascular risk factors. Most associations (all but WML vs CSF VEGF-D in patients with MCI) remained significant. Finally, we had only cross-sectional data on CSF biomarkers and could not study how these CSF biomarkers change dynamically in relation to cognition and WML.

There is a lack of validated fluid biomarkers for different aspects of vascular pathology in neurodegenerative diseases. Our findings strongly support CSF PlGF as a biomarker of vascular brain changes independent of AD copathology, which may have clinical importance. However, current results are not conclusive on how to use the studied biomarkers in clinical practice. Future studies may specifically test the additional value of CSF PlGF in the clinical management of patients with brain diseases and its use together with fluid biomarkers of other brain pathologies, for example, CSF Aβ42 and p-tau for AD. Truly longitudinal biomarker studies are needed to determine the role of CSF neuroinflammatory markers (especially PlGF) in relation to WML and other markers of SVD.

In conclusion, this study strengthens the role of CSF PlGF as a potential biomarker for WML, independent of AD pathology. In addition, we provide a comprehensive analysis on the relation between inflammatory markers and WML, showing that several proinflammatory markers and markers of angiogenesis are associated with WML. Surprisingly, there were inverse associations between some of the baseline measures of biomarkers and longitudinal WML changes, which need replication. Longitudinal analyses of cognition showed independent effects of CSF inflammatory markers and WML on longitudinal cognition, especially in people without cognitive impairment at baseline.

## Glossary

Aβ: β-amyloid
AD: Alzheimer disease
ADAS-cog: Alzheimer disease Assessments scale—cognitive subscale
BBB: blood-brain barrier
CDR: Clinical Dementia Rating
CU: cognitively unimpaired
FDR: false discovery rate
HCV: hippocampal volume
ICV: intracranial volume
IL: interleukin
IP-10: interferon γ–induced protein 10
LME: linear mixed effect
MCI: mild cognitive impairment
MCP-1: monocyte chemoattractant protein 1
MMSE: Mini-Mental State Examination
mPACC: modified preclinical Alzheimer composite score
PlGF: placental growth factor
SCD: subjective cognitive decline
sFlt-1: soluble fms-related tyrosine kinase 1
sICAM-1: soluble intercellular adhesion molecule 1
sVCAM-1: soluble vascular adhesion molecule 1
SVD: small vessel disease
TMTB: Trail Making Test B
VEGF: vascular endothelial growth factor
VEGFR1/VEGFR2: VEGF receptor 1/2
WML: white matter lesions
